# Neuromodulatory Effects of Transcranial Direct Current Stimulation on Motor Excitability in Rats

**DOI:** 10.1155/2019/4252943

**Published:** 2019-12-17

**Authors:** Hui-Hua Liu, Xiao-Kuo He, Hsin-Yung Chen, Chih-Wei Peng, Alexander Rotenberg, Chi-Hung Juan, Yu-Cheng Pei, Hao-Li Liu, Yung-Hsiao Chiang, Jia-Yi Wang, Xiao-Jun Feng, Ying-Zu Huang, Tsung-Hsun Hsieh

**Affiliations:** ^1^Department of Rehabilitation Medicine, Sun Yat-Sen Memorial Hospital, Sun Yat-Sen University, Guangzhou, China; ^2^School of Physical Therapy and Graduate Institute of Rehabilitation Science, Chang Gung University, Taoyuan, Taiwan; ^3^Department of Rehabilitation, The Fifth Hospital of Xiamen, Xiamen, Fujian, China; ^4^Department of Occupational Therapy and Institute of Behavioral Sciences, College of Medicine, Chang Gung University, Taoyuan, Taiwan; ^5^Department of Neurology and Dementia Center, Taoyuan Chang Gung Memorial Hospital, Taoyuan 33305, Taiwan; ^6^School of Biomedical Engineering, College of Biomedical Engineering, Taipei Medical University, Taipei, Taiwan; ^7^Department of Neurology, Boston Children's Hospital, Harvard Medical School, Boston, MA, USA; ^8^Institute of Cognitive Neuroscience, National Central University, Taoyuan, Taiwan; ^9^Brain Research Center, National Central University, Taoyuan, Taiwan; ^10^Department of Physical Medicine and Rehabilitation, Chang Gung Memorial Hospital, Taoyuan, Taiwan; ^11^Department of Electrical Engineering, Chang Gung University, Taoyuan, Taiwan; ^12^Department of Neurosurgery, Taipei Medical University Hospital, Taipei, Taiwan; ^13^Graduate Program on Neuroregeneration, Taipei Medical University, Taipei, Taiwan; ^14^Graduate Institute of Medical Sciences, College of Medical Science and Technology, Taipei Medical University, Taipei, Taiwan; ^15^Department of Rehabilitation Medicine, The Second Hospital of Anhui Medical University and Anhui Medical University, Hefei, China; ^16^Department of Neurology, Chang Gung Memorial Hospital and Chang Gung University College of Medicine, Taipei, Taiwan; ^17^Neuroscience Research Center, Chang Gung Memorial Hospital, Linkou, Taoyuan, Taiwan; ^18^Healthy Aging Research Center, Chang Gung University, Taoyuan, Taiwan

## Abstract

Transcranial direct current stimulation (tDCS) is a noninvasive technique for modulating neural plasticity and is considered to have therapeutic potential in neurological disorders. For the purpose of translational neuroscience research, a suitable animal model can be ideal for providing a stable condition for identifying mechanisms that can help to explore therapeutic strategies. Here, we developed a tDCS protocol for modulating motor excitability in anesthetized rats. To examine the responses of tDCS-elicited plasticity, the motor evoked potential (MEP) and MEP input-output (IO) curve elicited by epidural motor cortical electrical stimulus were evaluated at baseline and after 30 min of anodal tDCS or cathodal tDCS. Furthermore, a paired-pulse cortical electrical stimulus was applied to assess changes in the inhibitory network by measuring long-interval intracortical inhibition (LICI) before and after tDCS. In the results, analogous to those observed in humans, the present study demonstrates long-term potentiation- (LTP-) and long-term depression- (LTD-) like plasticity can be induced by tDCS protocol in anesthetized rats. We found that the MEPs were significantly enhanced immediately after anodal tDCS at 0.1 mA and 0.8 mA and remained enhanced for 30 min. Similarly, MEPs were suppressed immediately after cathodal tDCS at 0.8 mA and lasted for 30 min. No effect was noted on the MEP magnitude under sham tDCS stimulation. Furthermore, the IO curve slope was elevated following anodal tDCS and presented a trend toward diminished slope after cathodal tDCS. No significant differences in the LICI ratio of pre- to post-tDCS were observed. These results indicated that developed tDCS schemes can produce consistent, rapid, and controllable electrophysiological changes in corticomotor excitability in rats. This newly developed tDCS animal model could be useful to further explore mechanical insights and may serve as a translational platform bridging human and animal studies, establishing new therapeutic strategies for neurological disorders.

## 1. Introduction

Transcranial direct current stimulation (tDCS) is a noninvasive neuromodulation approach that can modulate motor cortical excitability with the application of a small current (1–2 mA) through scalp electrodes. Considerable research on tDCS has been conducted over the past decade, and this approach has also been studied in recent therapeutic studies on neurological and psychiatric conditions, such as stroke, Alzheimer's disease, and depression [1–4]. To assess changes in motor cortical excitability, transcranial magnetic stimulation (TMS) has generally been used to evoke motor evoked potentials (MEPs) that can be used to represent the excitability of the corticospinal pathway by measuring electromyography (EMG) in the targeted muscle [5–7]. Changes in motor cortical excitability induced by tDCS depend on polarity and duration. Generally, anodal tDCS enhances cortical excitability, whereas cathodal tDCS reduces it, as demonstrated by the amplitude of MEPs [8, 9]. Functional changes related to the alternation of cortical excitability induced by tDCS are considered to have therapeutic potential for human neurological and neuropsychological disorders, such as stroke, Parkinson's disease, and depression [10–12].

To enable translational research, in vivo experimental animal research might help clarify the mechanism of tDCS. In addition, to study physiological mechanisms, an animal model of tDCS can be ideal for providing a more stable condition to eliminate the discrepancy and clarify the existing effect. Using an animal model can also help explore effective therapeutic strategies for tDCS protocols. Although tDCS has been applied in some in vivo animal models, the polarity-dependent effects of tDCS on changes in motor cortical excitability have rarely been proved through MEPs in animal models, such as in rats [13, 14]. The major limitation of an animal model for recording MEPs is the low focalization because the TMS coil is considerably larger than the brain of small animals. To overcome this limitation, we developed a focused cortical electrical stimulation (CES) method by using epidural electrodes on the motor cortex in place of TMS in anesthetized rats for evoking MEPs [15, 16]. In this study, we developed a platform to test the polarity effects of tDCS on MEPs in rats based on the CES technique. We tested the modulation effect of anodal and cathodal tDCS on motor excitability by measuring changes in the size of MEPs. Furthermore, we applied the long-interval paired-pulse CES (LI-ppCES) scheme, mimicking paired-pulse TMS paradigms, to measure long-interval intracortical inhibition (LICI) and to identify whether tDCS can dynamically modulate inhibitory networks. The aim of this study was to develop a tDCS rat model as a translational platform to bridge physiological assessments between human and in vivo animal studies. We verified the model by assessing the polarity effects of tDCS on MEPs and further studied such effects on LICI in the motor cortex in rats. The platform built in this study can be helpful for identifying more detailed mechanisms underlying the neuromodulation effects of tDCS and enable more translational research in the tDCS rat model.

## 2. Materials and Methods

### 2.1. Animal Preparation

All experiments were conducted on 37 male adult Sprague-Dawley rats (body weight ranging from 350 to 450 g) obtained from the Laboratory Animal Center, Chang Gung University. To test the immediate effect of tDCS, rats were assigned to the following groups: sham tDCS (*n* = 8), low-dose anodal tDCS (0.1 mA; *n* = 8), high-dose anodal tDCS (0.8 mA; *n* = 8), low-dose cathodal tDCS (0.1 mA; *n* = 6), and high-dose cathodal tDCS (0.8 mA; *n* = 7). All animal procedures were conducted in accordance with the guidelines of the Institutional Animal Care and Use Committee at Chang Gung University. Procedures followed for measuring motor plasticity in the rat model were similar to those described in a previous study [15].

### 2.2. Implantation of tDCS and CES Electrodes

**Figure 1:**
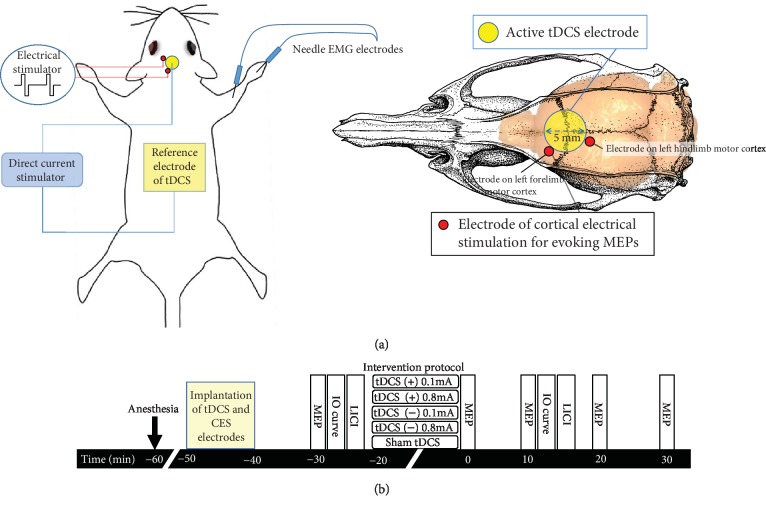
Placement and assembly of tDCS and CES electrodes. (a) Schematic diagram of the experimental design for testing changes in motor plasticity after tDCS in anesthetized rats. The center of epicranial tDCS tube electrodes is positioned at 0 mm left and 2.5 mm posterior to the bregma. A cortical epicranial electrode is fixed with dental cement. An active electrode is fixed to the skull with dental cement, and a reference electrode is placed into the abdominal region. Wires in the socket are wrapped to screw electrodes for eliciting motor evoked potentials (MEPs). (b) Following implantation of tDCS and CES electrodes, measurements of the MEP amplitude were assessed at baseline before tDCS and at every 10 min for up to 30 min after the end of tDCS. One block of the baseline input-output (IO) curve and one block of baseline long-interval intracortical inhibition (LICI) were recorded. After tDCS intervention, one block of the IO curve and one block of LICI were measured at 12 min and 15 min, respectively, after the end of tDCS intervention.

To implant the tDCS electrode, animals were deeply anesthetized for ~2.5 h with intraperitoneal injection (i.p.) of tiletamine-zolazepam (50 mg/kg; Zoletil, Vibac, France) and xylazine (10 mg/kg; Rompun, Bayer, Germany). Animals were then mounted on a stereotaxic frame. A 2 cm incision was made, and the implantation area was carefully cleared to expose the bregma line. To focally stimulate the motor cortex of rats, tDCS was applied from outside through an implantable round plastic socket (inner diameter: 5 mm). An active electrode was fixed to the skull with dental cement, and a reference electrode (5 cm × 3 cm, EASYpad™, Soterix Medical Inc., NY, USA) was placed into the abdominal region. A saline saturated sponge and an active electrode were inserted into the plastic tube during tDCS (Figure 1). Constant anodal or cathodal direct current of 0.1 mA or 0.8 mA (charge density: 0.13 mA/cm^2^ and 1.02 mA/cm^2^) was delivered to healthy rats for 20 min. Rats in the sham-control group did not receive any electrical stimulation.

To elicit MEPs, we implanted four epidural electrodes to perform CES. Burr holes were drilled for four stainless steel screw electrodes (1.6 mm diameter pole; E363, Plastics One Inc., Roanoke, VA) by using stereotactic coordinates. Cortical electrodes were placed epidurally into the primary motor cortex of the right forelimb (AP: 2.0 mm from the bregma; ML: 4.0 mm with respect to the midline) and hindlimb (AP: 3.0 mm from the bregma; ML: 1.25 mm with respect to the midline). Miniature sockets were then connected to two screw electrodes ipsilaterally through wires that were connected to an electrical stimulator (Model 2100, A-M Systems, Washington, USA). Recording of electromyographic (EMG) data was performed using 27G stainless steel needle electrodes (Axon Systems Inc., Hauppauge, NY, USA) inserted into the brachioradialis muscles bilaterally. The reference electrode was positioned distally in the paw. The ground electrode was inserted into the base of the tail of a rat [15]. The EMG signal was amplified (gain = 2000) and filtered using 60 Hz notch and 10–1000 Hz bandpass filters prior to digitization at 10 kHz (MP36, BIOPAC System, California, USA).

### 2.3. Assessment of Motor Plasticity

After impanation of tDCS and CES electrodes, the motor plasticity was then evaluated by changes in MEPs following different polarities of tDCS. The experimental design is illustrated in Figure 1(b). To reach a stable condition of anesthesia, the electrophysiological investigation was performed after 30 min anesthesia. Furthermore, to confirm no confounding effects of anesthesia, MEPs were recorded at several time points and the sham controlled tDCS group was used. The unilateral motor cortex was stimulated by CES and the contralateral magnitude of the evoked muscle contraction, which was quantified by EMG recorded through needle electrodes. Anodal tDCS (+) at 0.1 and 0.8 mA, cathodal tDCS (-) at 0.1 and 0.8 mA, and sham tDCS protocols were used to induce long-term potentiation- (LTP-), long-term depression- (LTD-) like changes, and the control condition, respectively. Biphasic single-pulse CES (amplitude: 1–10 V; pulse duration: 1 ms with 10 s intervals) was delivered to determine the resting motor threshold (RMT), which was defined as the intensity of electrical stimulation required to elicit peak-to-peak MEPs greater than 20 *μ*V in five out of 10 consecutive trials [15]. Baseline MEP was measured as the average of 12 MEPs evoked by a standard CES pulse delivered every 10 s at 120% RMT intensity. The 20 min tDCS protocol was then delivered to rats, and then, MEPs were assessed using the same single-pulse CES delivered in trains of 12 pulses, every 10 s for 2 min and then every 10 min until 30 min, after the end of tDCS (Figure 1(b)).

### 2.4. Input-Output Curve

To evaluate the relationship between CES intensity and the EMG response, an input-output (IO) curve, indicating the strength and integrity of corticospinal pathways and the MEP area as a function of stimulus intensity, was assessed before and after tDCS intervention. Compared with MEPs with single intensity of CES, the IO curve served as an extra index of excitability of large neuronal populations. To measure the IO curve, single CES pulse was applied at 90%, 100%, 110%, 120%, and 130% of RMT every 10 s for 5 times per stimulus intensity for 3 cycles. MEP amplitudes were measured and averaged for each stimulus intensity. To assess the effect of tDCS on the IO curve, IO curves were recorded before tDCS as a baseline level and at 12 min after the end of tDCS (Figure 1(b)).

### 2.5. Long-Interval Intracortical Inhibition

To measure LICI in rats, we modified our previously established TMS protocol from long-interval paired-pulse TMS (LI-ppTMS) to LI-ppCES [17]. In the LI-ppCES protocol, a conditioning stimulus at 120% RMT was delivered 200 ms before a succeeding CES test pulse of the same intensity. Ten pulse pairs were delivered every 10 s. LICI was assessed before tDCS and at 15 min after the end of tDCS (Figure 1(b)).

### 2.6. Data Analysis

The peak-to-peak amplitude of MEPs was measured for each recording session, and the averaged amplitude of 12 consecutive MEPs was automatically calculated using MATLAB (MathWorks, version 7.6, Natick, USA). To compare the effects of tDCS interventions on the MEP size, averaged MEP amplitudes for each time point were normalized to the averaged baseline amplitude of MEPs measured before tDCS. Data were further analyzed using SPSS for Windows version 17.0 (SPSS Inc., USA) with a significance level defined as *p* < 0.05. All data are presented as the mean ± standard error of the mean (SEM). To compare changes in MEP amplitudes with time (i.e., 0, 10, 20, and 30 min after tDCS) among five tDCS protocols (i.e., sham tDCS, 0.1 mA anodal tDCS, 0.8 mA anodal tDCS, 0.1 mA cathodal tDCS, and 0.8 mA cathodal tDCS), a two-way repeated measures analysis of variance (ANOVA) on MEP amplitudes normalized to the pre-tDCS baseline amplitude was applied. Unpaired *t*-tests were performed to compare groups at each time point when the main effect was significant in the group. A separate one-way ANOVA was used to examine the time course of changes in individual protocols on the absolute amplitude values of MEPs. Post hoc Fisher's LSD tests were used to compare between time points if needed.

For the analyses of IO curves in different polarities and intensities of tDCS, IO curves before and after tDCS were compared by performing a repeated measures ANOVA, including within-subject factors, time (before and after tDCS), and stimulus intensity (90%, 100%, 110%, 120%, and 130% RMT), separately for five tDCS protocols. An independent *t*-test was performed to compare between groups for each specific stimulus intensity.

LICI was expressed as a ratio of conditioned evoked MEP to unconditioned evoked MEP. The calculated ratios (conditioned MEP amplitude/unconditioned MEP amplitude) were averaged for each rat for each time point under different tDCS protocols. A two-way repeated measures ANOVA was used to compare the results of different protocols and time on LICI. Post hoc paired *t*-tests were used to compare pre- and post-LICI levels in each group.

## 3. Results

### 3.1. Effects of tDCS on MEPs

**Figure 2:**
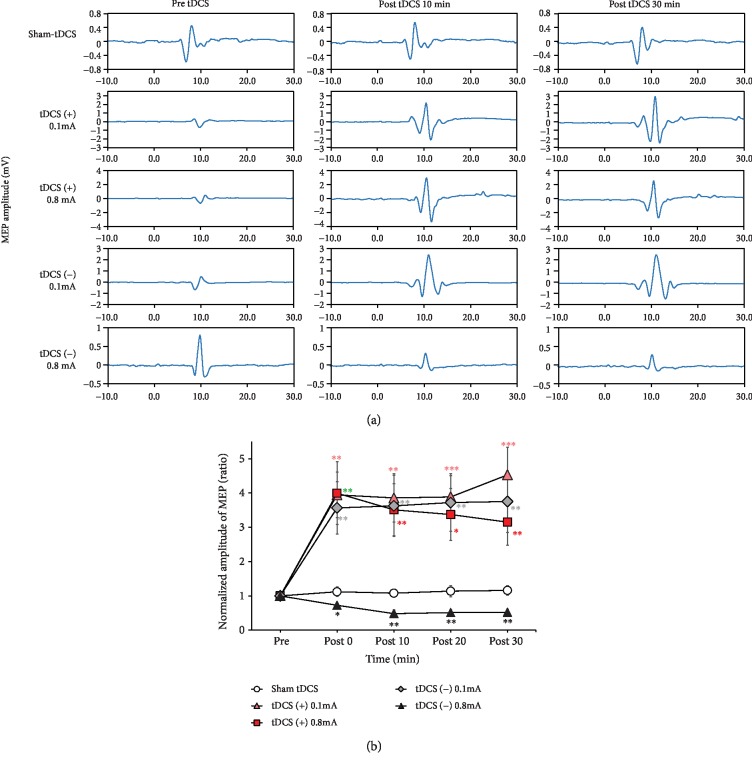
Effect of tDCS on MEPs in rats. (a) Time course changes of MEPs following sham, tDCS (+, 0.1 mA), tDCS (+, 0.8 mA), tDCS (-, 0.1 mA), and tDCS (-, 0.8 mA) interventions. Representative MEP traces following tDCS present no obvious change after sham stimulation, whereas traces of an increase in MEP amplitude after tDCS (+, 0.1 mA or 0.8 mA) and a reduction in MEP amplitude after tDCS (-, 0.8 mA) are observed. (b) Averaged changes in the MEP amplitude after sham, low-intensity anodal tDCS (0.1 mA), high-intensity anodal tDCS (0.8 mA), low-intensity cathodal tDCS (0.1 mA), and high-intensity cathodal tDCS (0.8 mA) are presented. Asterisks (∗) indicate a significant difference when compared with the sham group at the same time point (unpaired *t*-test). Error bars = SEM, ^∗^*p* < 0.05, ^∗∗^*p* < 0.01, ^∗∗∗^*p* < 0.001.

Representative changes in MEPs recorded pre-tDCS, 10 min post-tDCS, and 30 min post-tDCS are presented in Figure 2(a). An immediate increase in the MEP amplitude was observed over the course after low (0.1 mA) or high (0.8 mA) intensity anodal or low (0.1 mA) intensity cathodal tDCS was applied. A clear reduction in the MEP amplitude was observed at each measured time point following high-intensity (0.8 mA) cathodal tDCS. Time course MEP changes before and after different tDCS protocols are presented in Figure 2(b). The results of one-way ANOVA confirmed that MEPs did not change following sham tDCS (*F*_3,31_ = 0.056, *p* = 0.982). The findings of two-way repeated measures ANOVA presented significant main effects of the protocol (*F*_4,32_ = 7.23, *p* < 0.001) but no significant main effect of time (*F*_3,96_ = 0.81, *p* = 0.494) and protocol × time interaction (*F*_12,96_ = 1.35, *p* = 0.204). Compared with the sham tDCS group, subsequent post hoc Fisher's LSD test results demonstrated that MEPs were significantly enhanced immediately (post-tDCS 0 min, *p* < 0.05) and remained enhanced for up to 30 min (*p* < 0.01) in both anodal tDCS groups and in the low-intensity cathodal tDCS group (0.1 mA). MEPs decreased immediately (*p* < 0.05) and lasted for 30 min (*p* < 0.01) after high-intensity cathodal tDCS (Figure 2(b)).

### 3.2. Effects of tDCS on IO Curves

**Figure 3:**
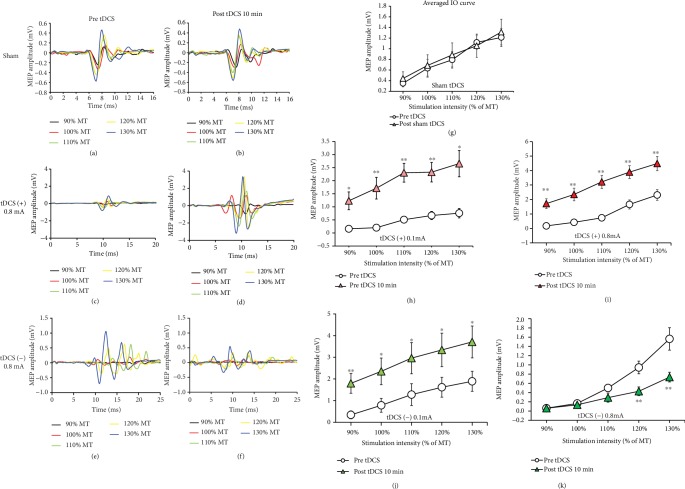
Sample of input-output (IO) recruitment curves for three tDCS conditions obtained before tDCS and after 10 min of tDCS (a–f). The IO curve is enhanced by 0.1 or 0.8 mA anodal tDCS and 0.1 mA cathodal tDCS (h–j), inhibited by 0.8 mA cathodal tDCS (k), and is unaltered by sham tDCS (g). Data are shown as the mean ± SEM. Asterisk (∗) denotes significance between pre- and post-tDCS at the same stimulation intensity (unpaired *t*-test, ^∗^*p* < 0.05, ^∗∗^*p* < 0.01).

Representative MEPs evoked by single-pulse CES at 90%–130% RMT pre- and post-sham, anodal tDCS (0.8 mA), and cathodal tDCS (0.8 mA) are presented in Figures 3(a)–3(f). In contrast to the sham tDCS group, which presented no change in the IO curve, the obtained averaged IO curves for anodal and cathodal tDCS groups under 0.1 mA or 0.8 mA stimulation presented significant differences from those obtained at the pre-tDCS stage. The results of two-way repeated measures ANOVA presented a significant main effect for stimulation intensity (*F*_1,14_ = 44.55, *p* < 0.001) but not for time (pre-tDCS and 10 min post-tDCS; *F*_1,14_ = 0.06, *p* = 0.807) and interaction between time and stimulation intensity (*F*_1,14_ = 0.02, *p* = 0.882). Statistical analysis performed on the IO curves before and after real tDCS intervention data revealed a significant main effect of time (*F*_1,14_ = 17.9, *p* = 0.001 in 0.1 mA anodal tDCS; *F*_1,14_ = 22.9, *p* < 0.001 in 0.8 mA anodal tDCS; *F*_1,10_ = 5.1, *p* = 0.048 in 0.1 mA cathodal tDCS; and *F*_1,12_ = 9.68, *p* = 0.009 in 0.8 mA cathodal tDCS) and intensity (*F*_4,56_ = 15.4, *p* < 0.001 in 0.1 mA anodal tDCS; *F*_4,56_ = 55.9, *p* < 0.001 in 0.8 mA anodal tDCS; *F*_4,40_ = 20.1, *p* < 0.001 in 0.1 mA cathodal tDCS; and *F*_4,48_ = 47.5, *p* < 0.001 in 0.8 mA cathodal tDCS), but no significant interaction between time and stimulation intensity (*F*_4,56_ = 2.35, *p* = 0.065 in 0.1 mA anodal tDCS; *F*_4,56_ = 1.8, *p* = 0.144 in 0.8 mA anodal tDCS; and *F*_4,40_ = 0.21, *p* = 0.932 in 0.1 mA cathodal tDCS) except under 0.8 mA cathodal tDCS (*F*_4,48_ = 7.5, *p* < 0.001). Further, for a comparison between MEP sizes at each intensity pre- and post-tDCS (Figures 3(g)–3(k)), independent *t*-tests with Bonferroni correction as post hoc tests were performed. These independent *t*-tests revealed a significant increase in MEP size after both low and high intensities of anodal tDCS and after a low intensity of cathodal tDCS (all *p* < 0.05), while MEPs were significantly suppressed at 120% and 130% RMT (*p* < 0.001) by high-intensity cathodal tDCS. No significant effect was found on the IO curve for before and after sham tDCS when sham stimulation was performed.

### 3.3. Effects of tDCS on LICI

**Figure 4:**
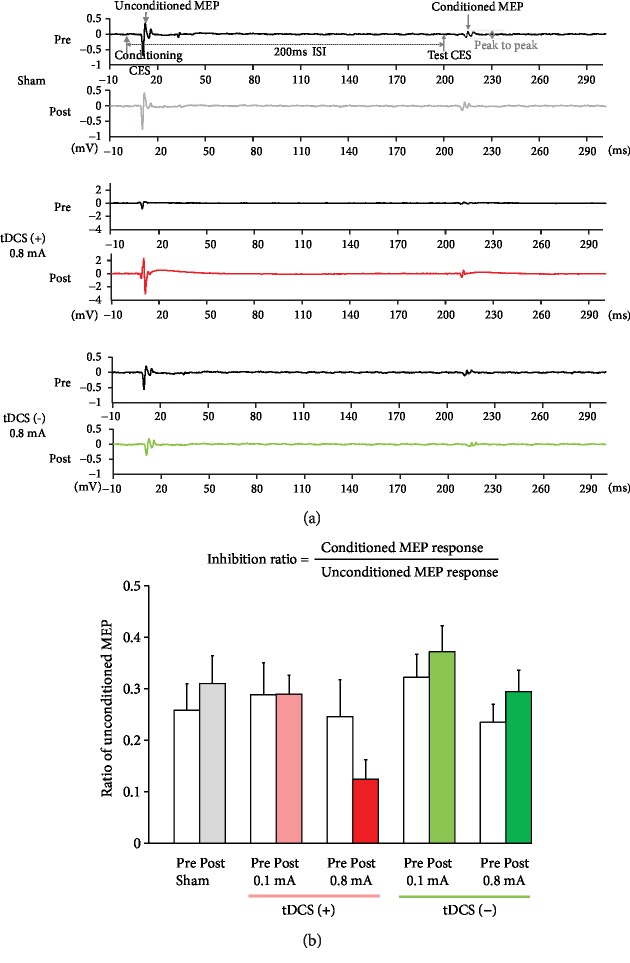
Effect of tDCS on LICI in rats. (a) MEP responses of long-interval paired-pulse cortical electrical stimulation (LI-ppCES) before and after sham, anodal tDCS (0.8 mA), and cathodal tDCS (0.8 mA) tests. (b) None of the tDCS protocols significantly altered LICI.

Application of the LI-ppCES protocol to an anesthetized rat led to the inhibition of MEP after the first CES. Examples of individual responses in LI-ppCES pre- and post-sham, anodal (0.8 mA), and cathodal tDCS (0.8 mA) are presented in Figure 4(a). The findings of two-way repeated measures ANOVA presented no significant protocol × time interaction (*F*_4,32_ = 1.87, *p* = 0.14) and main effects of the protocol (*F*_4,32_ = 1.71, *p* = 0.172) and time (*F*_1,32_ = 0.102, *p* = 0.752). The results of the paired *t*-test revealed no significant difference between LICI ratios for pre- and post-tDCS in the sham group (pre: 0.26 ± 0.14 vs. post: 0.31 ± 0.15; *p* = 0.11), anodal tDCS group (0.1 mA: pre: 0.29 ± 0.18 vs. post: 0.29 ± 0.10, *p* = 0.984; 0.8 mA: pre: 0.25 ± 0.20 vs. post: 0.12 ± 0.11, *p* = 0.067), and cathodal tDCS group (0.1 mA: pre: 0.32 ± 0.11 vs. post: 0.37 ± 0.12; *p* = 0.58; 0.8 mA: pre: 0.23 ± 0.12 vs. post: 0.29 ± 0.14; *p* = 0.35), indicating that anodal or cathodal tDCS did not modulate the strength of LICI (Figure 4(b)).

## 4. Discussion

In this study, we investigated the effects of different polarities of tDCS on motor cortical excitability and inhibition measured by CES-evoked MEPs in rats. Protocols commonly used for testing the effects of tDCS on motor cortical plasticity, IO curve, and LICI in the human brain were successfully translated into an in vivo tDCS rat model. To the best of our knowledge, this is the first study to demonstrate that tDCS modulates the measure of motor plasticity and LICI in MEPs in a polarity and dose-dependent manner in rats.

Studies have applied tDCS in animal models to identify the effects of tDCS on several aspects, such as cerebral blood flow [18], neurological disease models of stroke [19], epilepsy [20–22], depression [23], and pain [24]. Although the tDCS methodology has been reported in several animal studies, few studies have measured fundamental modulations in motor pathways after tDCS in a rat model. A previous study demonstrated the polarity effects of tDCS on MEPs, where MEPs were evoked by electric current that was delivered through same stimulation montage as tDCS [25]. In contrast to that study using the brain-thorax montage, we used a bipolar CES, which induced current similar to that by TMS in the cortex, to evoke MEPs in the current study [15]. The CES-MEP method provided a more focal stimulation than the brain-thorax montage for targeting the motor cortex and allowed better quantitative measurements of neuromodulation in motor excitability after tDCS [15, 16].

In the present study, anodal and cathodal tDCS were delivered at intensities of 0.13 mA/cm^2^ or 1.02 mA/cm^2^ which lie within the range of other animal studies that applied tDCS in rats [13, 20, 25] and have been considered safe. We established a tDCS-CES rat model to test motor plasticity induced by anodal or cathodal tDCS by measuring CES-evoked MEPs from the forelimb of rats. We found that polarity-dependent neuromodulation effects on the motor cortex were induced by different polarities of tDCS. We found that 20 min anodal tDCS at an intensity of 0.1 or 0.8 mA increased the size of MEPs for 30 min or more, whereas 20 min cathodal tDCS at an intensity of 0.8 mA decreased the size of MEPs for at least 30 min. These polarity-dependent results were analogous to those observed in humans [8, 26], indicating that this tDCS model may serve as a bridge between animal and human plasticity studies, at least for the motor pathway. In this study, no difference was observed between aftereffects induced by anodal stimulation at high (0.8 mA) and low intensities (0.1 mA) on cortical excitability, indicating that the effect anodal tDCS is not intensity sensitive in range we tested on motor cortical plasticity. This finding is in accordance with those of earlier human studies, which showed no stronger effects by increasing anodal intensities [26, 27]. By contrast, 20 min low-intensity cathodal tDCS (0.1 mA) did not inhibit MEPs but facilitated the amplitude of MEPs. Nonlinear intensity-dependent effects of cathodal tDCS have been reported in human studies [26, 28]. The optimal intensity of cathodal tDCS has been inconclusive. For example, Jamil et al. [26] systematically investigated intensity-dependent effects through MEPs and suggested that increasing cathodal intensities did not yield greater effects. They found that cathodal tDCS intensities of 0.5 mA and 1.0 mA led to excitability diminution, which was not achieved at 1.5 mA and 2.0 mA. By contrast, Batsikadze et al. [28] demonstrated that 20 min cathodal tDCS at an intensity of 2.0 mA shifted cortical plasticity from diminution to facilitation [28]. Our findings suggested that high-intensity cathodal tDCS (0.8 mA, 1.02 mA/cm^2^) follows the known polarity-dependent rule and exerts inhibitory effects on MEPs, whereas cathodal tDCS at a considerably low intensity (0.1 mA, 0.13 mA/cm^2^) may induce an opposite effect. Although the precise mechanism underlying the opposite effect remains unknown, results support the argument that the correlation between the effect and tDCS stimulation intensity, particularly cathodal, is not linear.

Our results revealed that the IO curve elevated significantly after anodal tDCS at intensities of 0.1 mA and 0.8 mA but decreased after cathodal tDCS at an intensity of 0.8 mA. These results fit well with a previous human tDCS study, which reported that the IO curve was dependent on tDCS polarity [29]. Compared with MEPs elicited by single-intensity CES, with the increased CES intensity to record MEPs, the IO curve serves as extra information to the excitability and recruitment of larger neuronal populations and can provide the global measures of corticospinal excitability. On the basis of previous human studies on the IO curve elicited by TMS, an increased or a decreased slope of the IO curve can be due to neuronal membrane excitability or synaptic mechanisms, which are controlled by sodium channel blockers or neurotransmitters [30, 31]. In this study, we also provided an animal experimental model, which can be useful for further identification of mechanical insights of tDCS. Additionally, we found that the IO curve totally shifted and elevated after anodal tDCS. This parallel shifting pattern suggested that RMT was decreased by anodal tDCS and sodium channels were involved in the facilitatory effect of the anodal stimulation on MEPs.

In addition, we examined whether tDCS modulates intracortical inhibition when cortical excitability is altered. We found that both anodal and cathodal tDCS failed to modulate the strength of LICI. Paired-pulse TMS protocols have been commonly used to examine short- and long-interval intracortical inhibition within the motor cortex (SICI and LICI), which are presumably mediated by gamma-aminobutyric acid A (GABA_A_) and GABA_B_ receptors, respectively [32, 33]. SICI has been known to be reduced by anodal tDCS but enhanced by cathodal tDCS, whereas LICI has been found to be unaffected by tDCS in human studies [29, 32, 34, 35]. To the best of our knowledge, the present study is the first to examine the effect of tDCS on LICI in an animal model. The results are in line with those of human studies, suggesting that tDCS does not modulate GABA_B_-mediated circuits involving LICI.

## 5. Conclusion

We set up a CES scheme for testing tDCS-induced motor plasticity and the effect of tDCS on LICI by measuring MEPs in vivo in rats. Results, which showed polarity-dependent modulation effects of tDCS on MEPs, confirmed that this tDCS-CES rat model successfully mimicked the situation in humans. Thus, this model can be used for translating tDCS studies from humans to rats and vice versa. This newly developed tDCS-CES rat model would be useful for studying the mechanism of tDCS-induced plasticity and for developing therapeutic strategies with tDCS for neurological and neuropsychological disorders.

## Data Availability

The data generated and analyzed during the current study are available from the corresponding authors on reasonable request.

## References

[1] Stagg C. J., Nitsche M. A. (2011). Physiological basis of transcranial direct current stimulation. *Neuroscientist*.

[2] Brunoni A. R., Nitsche M. A., Bolognini N. (2012). Clinical research with transcranial direct current stimulation (tDCS): challenges and future directions. *Brain Stimulation*.

[3] Zimerman M., Heise K. F., Hoppe J., Cohen L. G., Gerloff C., Hummel F. C. (2012). Modulation of training by single-session transcranial direct current stimulation to the intact motor cortex enhances motor skill acquisition of the paretic hand. *Stroke*.

[4] Palm U., Hasan A., Strube W., Padberg F. (2016). tDCS for the treatment of depression: a comprehensive review. *European Archives of Psychiatry and Clinical Neuroscience*.

[5] Pascual-Leone A., Tormos J. M., Keenan J., Tarazona F., Cañete C., Catalá M. D. (1998). Study and modulation of human cortical excitability with transcranial magnetic stimulation. *Journal of Clinical Neurophysiology*.

[6] Kobayashi M., Pascual-Leone A. (2003). Transcranial magnetic stimulation in neurology. *The Lancet Neurology*.

[7] Huang Y. Z., Chen R. S., Rothwell J. C., Wen H. Y. (2007). The after-effect of human theta burst stimulation is NMDA receptor dependent. *Clinical Neurophysiology*.

[8] Nitsche M. A., Paulus W. (2000). Excitability changes induced in the human motor cortex by weak transcranial direct current stimulation. *The Journal of Physiology*.

[9] Nitsche M. A., Paulus W. (2001). Sustained excitability elevations induced by transcranial DC motor cortex stimulation in humans. *Neurology*.

[10] Pollock A., Farmer S. E., Brady M. C. (2013). Interventions for improving upper limb function after stroke. *Cochrane Database of Systematic Reviews*.

[11] Bennabi D., Pedron S Ã.¨n., Haffen E., Monnin J., Peterschmitt Y., Van Waes V. (2014). Transcranial direct current stimulation for memory enhancement: from clinical research to animal models. *Frontiers in Systems Neuroscience*.

[12] Mondino M., Bennabi D., Poulet E., Galvao F., Brunelin J., Haffen E. (2014). Can transcranial direct current stimulation (tDCS) alleviate symptoms and improve cognition in psychiatric disorders?. *World Journal of Biological Psychiatry*.

[13] Brunoni A. R., Fregni F., Pagano R. L. (2011). Translational research in transcranial direct current stimulation (tDCS): a systematic review of studies in animals. *Reviews in the Neurosciences*.

[14] Jackson M. P., Rahman A., Lafon B. (2016). Animal models of transcranial direct current stimulation: methods and mechanisms. *Clinical Neurophysiology*.

[15] Hsieh T. H., Huang Y. Z., Chen J. J. J. (2015). Novel use of theta burst cortical electrical stimulation for modulating motor plasticity in rats. *Journal of Medical and Biological Engineering*.

[16] Wu C. W., Chiu W. T., Hsieh T. H., Hsieh C. H., Chen J. J. J. (2018). Modulation of motor excitability by cortical optogenetic theta burst stimulation. *PLoS One*.

[17] Vahabzadeh-Hagh A. M., Muller P. A., Pascual-Leone A., Jensen F. E., Rotenberg A. (2011). Measures of cortical inhibition by paired-pulse transcranial magnetic stimulation in anesthetized rats. *Journal of Neurophysiology*.

[18] Wachter D., Wrede A., Schulz-Schaeffer W. (2011). Transcranial direct current stimulation induces polarity-specific changes of cortical blood perfusion in the rat. *Experimental Neurology*.

[19] Kim S. J., Kim B. K., Ko Y. J., Bang M. S., Kim M. H., Han T. R. (2010). Functional and histologic changes after repeated transcranial direct current stimulation in rat stroke model. *Journal of Korean Medical Science*.

[20] Liebetanz D., Klinker F., Hering D. (2006). Anticonvulsant effects of transcranial direct-current stimulation (tDCS) in the rat cortical ramp model of focal epilepsy. *Epilepsia*.

[21] Dhamne S. C., Ekstein D., Zhuo Z. (2015). Acute seizure suppression by transcranial direct current stimulation in rats. *Annals of Clinical and Translational Neurology*.

[22] Kamida T., Kong S., Eshima N., Abe T., Fujiki M., Kobayashi H. (2011). Transcranial direct current stimulation decreases convulsions and spatial memory deficits following pilocarpine-induced status epilepticus in immature rats. *Behavioural Brain Research*.

[23] Fregni F., Liebetanz D., Monte-Silva K. K. (2007). Effects of transcranial direct current stimulation coupled with repetitive electrical stimulation on cortical spreading depression. *Experimental Neurology*.

[24] Nekhendzy V., Fender C. P., Davies M. F. (2004). The antinociceptive effect of transcranial electrostimulation with combined direct and alternating current in freely moving rats. *Anesthesia and Analgesia*.

[25] Cambiaghi M., Velikova S., Gonzalez-Rosa J. J., Cursi M., Comi G., Leocani L. (2010). Brain transcranial direct current stimulation modulates motor excitability in mice. *European Journal of Neuroscience*.

[26] Jamil A., Batsikadze G., Kuo H.-I. (2017). Systematic evaluation of the impact of stimulation intensity on neuroplastic after-effects induced by transcranial direct current stimulation. *The Journal of Physiology*.

[27] Kidgell D. J., Daly R. M., Young K. (2013). Different current intensities of anodal transcranial direct current stimulation do not differentially modulate motor cortex plasticity. *Neural Plasticity*.

[28] Batsikadze G., Moliadze V., Paulus W., Kuo M.‐. F., Nitsche M. A. (2013). Partially non-linear stimulation intensity-dependent effects of direct current stimulation on motor cortex excitability in humans. *The Journal of Physiology*.

[29] Nitsche M. A., Seeber A., Frommann K. (2007). Modulating parameters of excitability during and after transcranial direct current stimulation of the human motor cortex. *Clinical Neurophysiology*.

[30] Ziemann U. (2013). Pharmaco-transcranial magnetic stimulation studies of motor excitability. *Handbook of Clinical Neurology*.

[31] Ziemann U., Lonnecker S., Steinhoff B. J., Paulus W. (1996). Effects of antiepileptic drugs on motor cortex excitability in humans: a transcranial magnetic stimulation study. *Annals of Neurology*.

[32] Tremblay S., Beaule V., Lepage J. F., Theoret H. (2013). Anodal transcranial direct current stimulation modulates GABAB-related intracortical inhibition in the M1 of healthy individuals. *Neuroreport*.

[33] Ziemann U., Lönnecker S., Steinhoff B. J., Paulus W. (1996). The effect of lorazepam on the motor cortical excitability in man. *Experimental Brain Research*.

[34] Antal A., Terney D., Kuhnl S., Paulus W. (2010). Anodal transcranial direct current stimulation of the motor cortex ameliorates chronic pain and reduces short intracortical inhibition. *Journal of Pain and Symptom Management*.

[35] Hummel F. (2005). Effects of non-invasive cortical stimulation on skilled motor function in chronic stroke. *Brain*.

